# Lectin-Based Immunophenotyping and Whole Proteomic Profiling of CT-26 Colon Carcinoma Murine Model

**DOI:** 10.3390/ijms25074022

**Published:** 2024-04-04

**Authors:** Anna Faragó, Ágnes Zvara, László Tiszlavicz, Éva Hunyadi-Gulyás, Zsuzsanna Darula, Zoltán Hegedűs, Enikő Szabó, Sára Eszter Surguta, József Tóvári, László G. Puskás, Gábor J. Szebeni

**Affiliations:** 1 Astridbio Technologies Ltd., Wimmer Fülöp utca 1, H6728 Szeged, Hungary; a.farago@astridbio.com; 2 University of Szeged, Albert Szent-Györgyi Medical School, Doctoral School of Multidisciplinary Medical Sciences, Dóm tér 9, H6720 Szeged, Hungary; 3 Institute of Genetics, Laboratory of Functional Genomics, HUN-REN Biological Research Centre, Temesvári krt. 62, H6726 Szeged, Hungary; zvara.agnes@brc.hu (Á.Z.); szabo.eniko@brc.hu (E.S.); 4 Core Facility HUN-REN Biological Research Centre, Temesvári krt. 62, H6726 Szeged, Hungary; gulyas.eva@brc.hu (É.H.-G.); darula.zsuzsanna@brc.hu (Z.D.);; 5 Department of Pathology, University of Szeged, Állomás u. 2, H6725 Szeged, Hungary; tiszlavicz.laszlo@med.u-szeged.hu; 6 Laboratory of Proteomics Research, HUN-REN Biological Research Centre, Temesvári krt. 62, H6726 Szeged, Hungary; 7 The Hungarian Centre of Excellence for Molecular Medicine (HCEMM) Single Cell Omics Advanced Core Facility, Biological Research Centre, Temesvári krt. 62, H6726 Szeged, Hungary; 8 Laboratory of Bioinformatics, HUN-REN Biological Research Centre, Temesvári krt. 62, H6726 Szeged, Hungary; 9 Department of Biochemistry and Medical Chemistry, Medical School, University of Pécs, Szigeti út 12, H7624 Pécs, Hungary; 10 Department of Experimental Pharmacology, The National Tumor Biology Laboratory, National Institute of Oncology, Ráth György u. 7-9, H1122 Budapest, Hungary; surguta.sara@ext.oncol.hu (S.E.S.); tovari.jozsef@oncol.hu (J.T.); 11 Avidin Ltd., Alsó Kikötő sor 11/D, H6726 Szeged, Hungary; 12 Avicor Ltd., Alsó Kikötő sor 11/D, H6726 Szeged, Hungary; 13 Department of Internal Medicine, Hematology Centre, Faculty of Medicine University of Szeged, H6725 Szeged, Hungary

**Keywords:** colorectal carcinoma, lectin binding sugar code, proteomic analysis of murine CRC

## Abstract

A murine colorectal carcinoma (CRC) model was established. CT26 colon carcinoma cells were injected into BALB/c mice’s spleen to study the primary tumor and the mechanisms of cell spread of colon cancer to the liver. The CRC was verified by the immunohistochemistry of Pan Cytokeratin and Vimentin expression. Immunophenotyping of leukocytes isolated from CRC-bearing BALB/c mice or healthy controls, such as CD19+ B cells, CD11+ myeloid cells, and CD3+ T cells, was carried out using fluorochrome-labeled lectins. The binding of six lectins to white blood cells, such as galectin-1 (Gal1), siglec-1 (Sig1), *Sambucus nigra* lectin (SNA), *Aleuria aurantia* lectin (AAL), *Phytolacca americana* lectin (PWM), and galectin-3 (Gal3), was assayed. Flow cytometric analysis of the splenocytes revealed the increased binding of SNA, and AAL to CD3 + T cells and CD11b myeloid cells; and increased siglec-1 and AAL binding to CD19 B cells of the tumor-bearing mice. The whole proteomic analysis of the established CRC-bearing liver and spleen versus healthy tissues identified differentially expressed proteins, characteristic of the primary or secondary CRC tissues. KEGG Gene Ontology bioinformatic analysis delineated the established murine CRC characteristic protein interaction networks, biological pathways, and cellular processes involved in CRC. Galectin-1 and S100A4 were identified as upregulated proteins in the primary and secondary CT26 tumor tissues, and these were previously reported to contribute to the poor prognosis of CRC patients. Modelling the development of liver colonization of CRC by the injection of CT26 cells into the spleen may facilitate the understanding of carcinogenesis in human CRC and contribute to the development of novel therapeutic strategies.

## 1. Introduction

Colorectal cancer (CRC) is the second leading cause of death and the third most common cancer in both men and women worldwide [1,2]. Even though the early stages of colon cancer are curable, CRC is often ignored because of atypical clinical symptoms. In addition, around 50% of people who have colorectal cancer do not report any symptoms at all [3]. Studies have shown that 25–30% of patients with CRC develop liver metastases and have poor prognosis [4]. Therefore, experimental animal models have an important role in determining the pathogenesis of CRC, investigating the development of possible metastasis, and testing new drug candidates. Mouse CRC models performing tumor induction, growth, and spread are best suited to study the biology of tumors and are also suitable for understanding tumor-related immune activation. The tumor microenvironment and the precise significance of the connection with immune surveillance are still not fully understood [5]. Tumor infiltration of immune cells is associated with a different prognosis. For example, a high density of infiltrating T, B, and NK cells is associated with a good prognosis [6,7,8]. However, M2-type macrophages and T regulatory cells were connected to tumor progression and consequently poor prognosis [9,10,11]. Thus, understanding the immune microenvironment can improve novel diagnostic tools and may predict a more precise prognosis. CT26 is a hypermutated murine colorectal carcinoma cell line that is the most commonly used preclinical model for CRC tumors [12]. BALB/c-derived CT26 is a well-characterized, cost-effective cell-line model, which was developed earlier by others; they exposed mice to N-nitroso-N-methylurethane (NMU) resulting in a rapidly growing colorectal carcinoma that was cloned, selected, and named CT26 [13]. To establish this cancer model, CT26 colon carcinoma cells were injected into BALB/c mice’s spleen in our current work. The injection of tumor cells into the spleen and forming an artificial primary tumor location represent ideal tools to study the mechanisms of cell spread, a colonization of colon cancer to the liver. Therefore, this method is suitable for examining both primary spleen tumors and possible secondary or metastatic liver tumors [14]. We aimed to develop a glycosylation pattern-based analysis of isolated peripheral white blood cells and isolated spleen cells by fluorescent dye-labeled lectins for multicolor flow cytometry. Immune evasion, thus alterations in the function of the immune system, is key to cancer progression as well as later during metastasis, but the immune cell’s surface glycosylation role in cancer diagnosis or prognosis is not completely defined in CRC. Here, we demonstrate a potential lectin-based screening of CRC using multi-color flow cytometry analyses of peripheral blood and spleen cells. Lectins are carbohydrate-binding proteins with a certain specificity for unique sugar moieties. We used this lectin-specific binding ability to map individual cell surface patterns. Similarly to all other cells, the immune cells sense environmental signals and express cell surface-associated glycoproteins and glycolipids. The glycosylation pattern is critical for physiological and pathological cellular functions; thus, an altered glycosylation pattern can modulate immunological responses, such as inflammation and immune response to cancer [15]. Specific fluorescent dye-labeled carbohydrate-binding lectins could be a novel tool for cancer diagnosis. Fluorescence Activated Cell Sorting (FACS) is a rapid technology for the analysis of single cells in a solution that provides fast, multi-parametric, and quantitative recording of fluorescent signals from individual cell populations [16]. Cell surface glycan alterations in cancer have been widely documented [17,18]. Fucosylation is generally increased during carcinogenesis. Chen et al. reported the overexpression of fucosyltransferase 8 (FUT8) in non-small cell lung cancer (NSCLC) [17]. In colon carcinoma (CRC), enzymes that participate in fucosylation show upregulated expression (13). Therefore, *Aleuria aurantia* lectin (AAL), recognizing α 1-6, α 1-3, and α 1-4 fucose moieties, has been studied in our current work. Furthermore, terminal sialylation of glycan chains is widely known as a prominent alteration in most types of solid cancers [19]. Sialic acid-binding Ig-like lectins (siglecs) are expressed on the immune cell’s surface. Altered sialylation of tumor cells may interact with siglecs of the immune cells and may promote immune system evasion [20]. Therefore, we chose human siglec-1 lectin with α 2-3 sialic acid and *Sambucus nigra* lectin (SNA) with α 2-6 sialic acid specificity to investigate. SNA is known for the detection of colorectal cancer from plasma with high specificity [21]. Galectin-1 (Gal1) and galectin-3 (Gal3) are reported to be overexpressed in hepatocellular carcinoma (HCC). Gal1 correlates with tumor aggressiveness and metastases and Gal3 promotes angiogenesis and inhibits cancer cell apoptosis [22,23]. Galectins have the preferred specificity of N-Acetyllactosamine (LacNAc) residues and aberrant expression of LacNAc is reported as a characteristic of gastric cancer (GC), and LacNAc may also serve as a scaffold for further modification with sialic acid or fucose moieties [24]. So, we studied Gal1 and Gal3 binding in our current study. Finally, we chose N-acetylglucosamine (GlcNAc) binding *Phytolacca americana* lectin (Pokeweed, PWM) to study because N-acetylglucosaminyltransferase V (GnT-V) is known for its upregulation in metastatic cancer which catalyzes the formation of GlcNAc moieties [25]. Furthermore, HSP90b and Annexin A1 proteins were found to be GlcNAcylated in colon cancer tissues [26].

One of our further aims was the extensive proteomic analysis of primary spleen and metastatic liver tumors after 3 weeks of establishment. We performed Liquid Chromatography Mass Spectrometry (LC-MS) on appropriately sized tumors to identify and quantify the proteome of each tumor tissue. After the present protein identification, we quantified the abundance of proteins in the tumors and found significant differences in primary and metastatic tumor tissue compared to healthy control tissues. A bioinformatic analysis of proteomic data showed the interactive networks among proteins and their role in biological processes and pathways with special focus on enzymes involved in glycosylation.

## 2. Results

### 2.1. Establishment of Murine CRC

**Figure 1:**
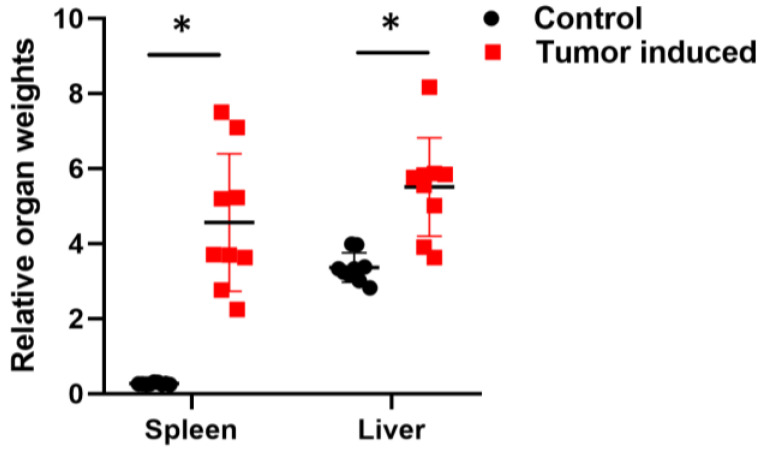
Calculated relative organ weights to the brain. Relative spleens and liver weights with CRC showed significant changes compared with healthy relative organ weights. To calculate the relative weight, the given organ weight was divided by the weight of the brain. Arithmetic mean values and standard deviations (error bars) are shown, (n = 9 CT 26-bearing; n = 9 control). Statistical analysis was performed using a two-tailed, heteroscedastic Student’s *t*-test to evaluate the statistical significance (set at * *p* < 0.05) between two given experimental groups: pairwise comparison of each sample.

Monitoring the animals has an important role in following tumor growth and choosing the right time for euthanasia. Animal bodyweight monitoring is not a sufficient procedure in studies with tumor growth because of the significative tumor mass. Although the mice show weight loss, this is not always evident due to the weight of the tumors [27]. Tumor-bearing mice frequently lose their body weight during tumor progression (cachexia), but the weight of the brain remains almost constant. Therefore, calculating relative organ weights to the brain gives a more detailed picture of cancer progression (Figure 1) [28,29].

**Figure 2:**
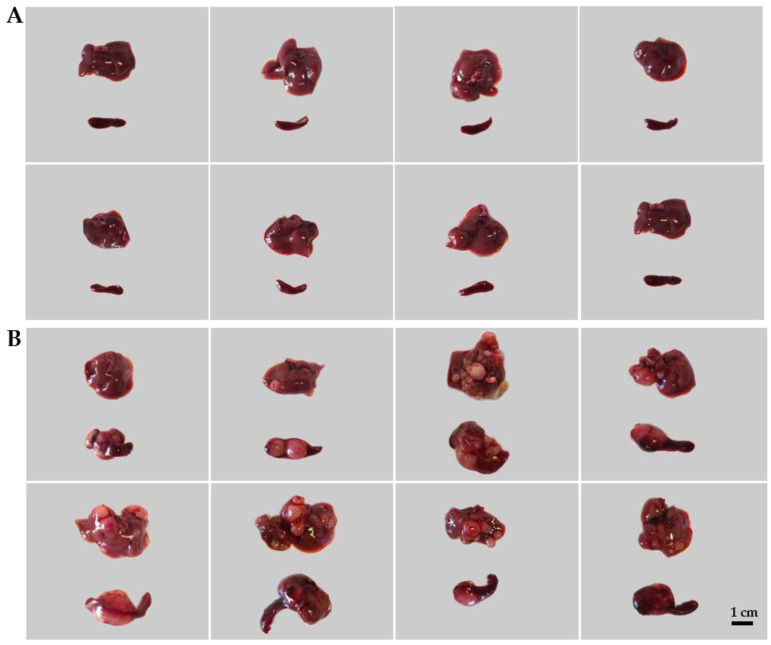
The induction of colorectal carcinoma (CRC) in mice. Representative images of healthy livers and spleens (**A**: control) compared with spleens and livers with CRC (**B**: CT26-bearing). The induction of the CRC model is described in the Materials and Methods Section 4.2 and Section 4.3.

The treated animals were sacrificed on day 22 after tumor inoculations. Tumors were evaluated macroscopically (Figure 2) and processed further for immunohistochemistry (IHC). Leukocytes and spleen cells were isolated as described in the Material and Methods Section 4.5 and Section 4.6.

**Figure 3:**
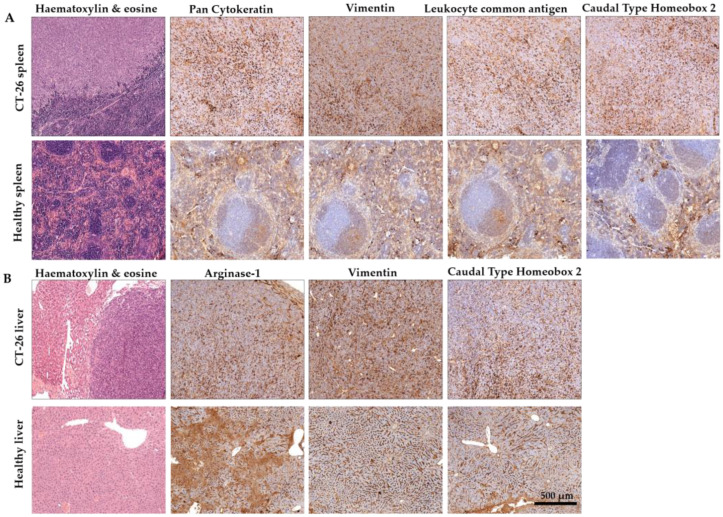
Representative immunohistochemistry images of the spleens (**A**) and the liver (**B**) of murine colorectal carcinomas (CRC). (**A**) Pan Cytokeratin, where Vimentin positive staining confirmed that spleen-injected CT26 colon carcinoma is an adenocarcinoma probably dedifferentiated in the stem cell direction with definite EMT. (**B**) The secondary liver cancer was verified by Vimentin staining. ((**A**): tumor-induced animal’s spleen and healthy spleen; (**B**): tumor-induced animal’s liver and healthy liver, scale bar 500 μm; magnification scale: 10×).

Colorectal carcinoma was verified by IHC combined with Hematoxylin and Eosin staining (Figure 3). Cytokeratins are structural proteins and their expression is maintained in malignant transformation [30]. Pan-Cytokeratin has been reported to stain the carcinomas (epithelial tumor cells) and it highlights tumor cell budding (detachment) at the invasive front [31,32].Vimentin is expressed in mesenchymal cells and correlated with the malignancy of cancer cells [33]. Vimentin is known for its effects on colorectal cancer proliferation, invasion, and migration, and vimentin expression is associated with higher tumor grade, metastasis, and shorter survival in colorectal cancer [34,35]. Vimentin is also a marker of epithelial–mesenchymal transition (EMT) in CRC [34]. CD45 (leukocyte common antigen) is another marker of EMT. The expression of CD45 in CRC can be induced after chemotherapy in the EpCAM + cancer stem-like cells (CSCs), which collectively enhances stemness and may contribute to chemoresistance [36]. Caudal-type homeobox 2 (CDX2) is an intestine-specific transcription factor and it functions as a tumor suppressor [37,38]. Therefore, the loss of nuclear CDX2 expression is a useful diagnostic marker and suggests a poor prognosis of CRC [39]. Arginase-1 has been reported as an indicator of poor prognosis in CRC [40] and a marker of non-malignant hepatocytes and hepatocellular carcinoma [41,42]; therefore, it was assayed in liver metastasis. In our model, the spleen-injected murine colorectal carcinoma is an adenocarcinoma probably dedifferentiated in the stem cell direction with definite EMT (Figure 3A). The closest organ to the primary tumor in our model was the liver. IHC verified the expression of Arginase-1, Vimentin, and CDX-2 in the liver metastatic tissues (Figure 3B).

### 2.2. Flow Cytometric Immunophenotyping of the Leukocytes in CRC-Bearing Mice Using Fluorescently Labeled Lectins

**Figure 4:**
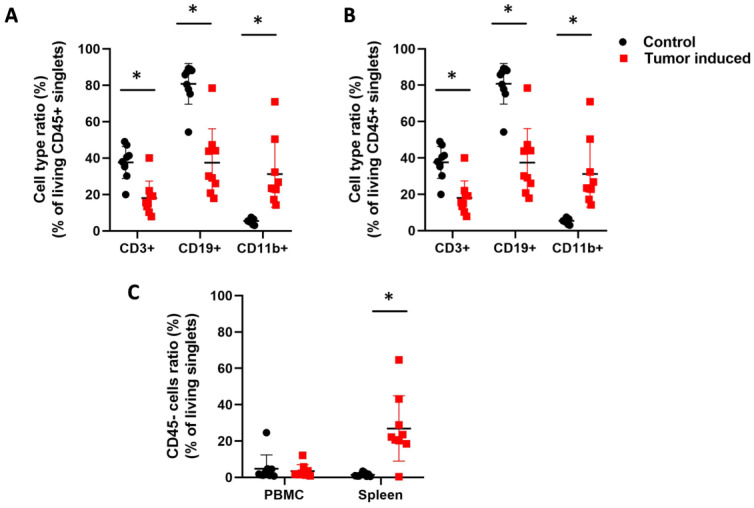
Flow cytometric analysis of the leukocytes. CD45+ white blood cells were assayed from the blood (**A**) and spleen (**B**) about the parental ratio of CD3+ T cells, CD11b+ myeloid cells, and CD19+ B cells in healthy controls versus CRC-bearing mice. (**C**) The immature CD45 non-adherent cells were assayed from the PBMC and spleen. The CT26-bearing mice showed an expansion of immature leukocytes in the spleen. Arithmetic mean values and standard deviations (error bars) are shown, (n = 9 CT 26-bearing, n = 9 control). For flow cytometry, statistical analysis was performed using two-tailed, heteroscedastic Student’s *t*-test to evaluate the statistical significance (set at * *p* < 0.05) between two given experimental groups: pairwise comparison of each sample.

The immunophenotyping was carried out using fluorochrome-labeled lectins and anti-CD3, anti-CD11b, and anti-CD19 antibodies to define T cells, myeloid cells, and B cells, respectively. Therefore, the ratio of the main leukocyte populations was also investigated in CRC (Figure 4). Induced CRC flow cytometry analysis showed that CD3+ T cells, CD19+ B cells, and CD11b+ myeloid cells changed similarly in the case of peripheral blood cells and splenocytes. CD3+ T cell count was significantly decreased in CRC mice compared to the healthy control animals (arithmetic mean ± SD of the parental %; PBMC; 14.8 ± 5.8 vs. 22.9 ± 7.5, *p* = 0.02 (Figure 4A); spleen; 18 ± 9.3 vs. 37.5 ± 8.8, *p* = 3.2 × 10^−4^ (Figure 4B). Tumor-induced immunosuppression via T cells is a well-known phenomenon in cancer progression [43]. Also, CD19+ B cells showed a decreased number in two sample types, both in PBMCs and in the spleen (PBMC; 17.8 ± 10.4 vs. 38.6 ± 19.2, *p* = 0.01 (Figure 4A); spleen; 37.5 ± 18.6 vs. 80.7 ± 11.1, *p* = 1.9 × 10^−5^ (Figure 4B). Low numbers of CD19+ B cells can be a sign of general B cell lymphocytopenia, which is a cellular immunodeficiency in immunologically stressful situations [44]. However, CD11b+ myeloid cells showed a significantly increased ratio compared to the healthy controls (PBMC; 45.8 ± 12.3 vs. 31 ± 12.2, *p* = 0.02 (Figure 4A) spleen; 31.2 ± 18.2 vs. 5.4 ± 1.4, *p*= 6.3 × 10^−4^ (Figure 4B). Massive splenomegaly is a possible explanation for an increased number of myeloid cells predominated by granulocytic cells. Splenomegaly is one sign of myeloid cell expansion due to cancer-related inflammation in tumor-bearing hosts [45]. Significantly increased CD45-cell count of splenocytes (26.9 ± 17.9 vs. 1.4 ± 1, *p* = 6.2 × 10^−4^) could support the expansion of immature leukocytes (Figure 4C).

**Figure 5:**
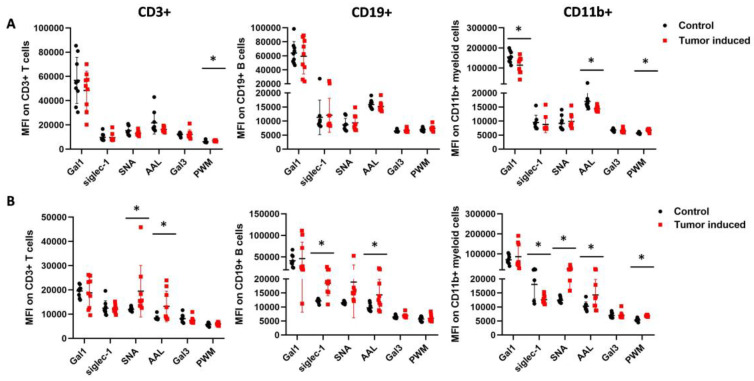
Flow cytometric analysis of the leukocytes from the blood (**A**) and spleen (**B**) regarding the lectin binding median intensity of CD3+ T cells, CD11b+ myeloid cells, and CD19+ B cells in healthy controls versus CRC-bearing mice. The spleens were excised from healthy control and CRC-bearing mice, and leukocytes from the spleen and the blood were incubated with fluorescently labeled antibodies or lectins. The binding of AAL was significantly higher in the mouse’s spleen with induced CRC versus the healthy control. However, the binding of AAL to PBMCs was significantly lower. The binding of SNA to the sialic acid moiety was significantly higher on the CD3+ T cells and the CD11b+ myeloid cells surface of CRC-bearing mice’s spleen cells vs. controls. Also, PWM lectin showed significant differences in binding to cell surfaces. Arithmetic mean values and standard deviations (error bars) are shown, (n = 9 CT 26-bearing, n = 9 control). For flow cytometry statistical analysis was performed using two-tailed, heteroscedastic Student’s *t*-test to evaluate the statistical significance (set at * *p* < 0.05) between two given experimental groups: pairwise comparison of each sample.

The binding of six lectins to immune cells, such as galectin-1 (Gal1), siglec-1 (Sig1), *Sambucus nigra* lectin (SNA), *Aleuria aurantia* lectin (AAL), *Phytolacca americana* lectin (PWM), and galectin-3 (Gal3), was investigated. There were significant differences in MFI (median fluorescence intensity) in the binding of the lectins to three different types of immune cells of the CRC-bearing mice compared to healthy controls (Figure 5). The binding of AAL to fucose/arabinose structures was significantly higher on the cell surface of all three immune cell types of the mouse’s spleen with induced CRC versus the healthy control (mean ± SD of the median fluorescence intensity: CD3+: 13,183 ± 6309 vs. 8392 ± 952, *p* = 0.03; CD11b+: 14,271 ± 5582 vs. 10,298 ± 1516, *p*= 0.05; CD19+: 14,331 ± 5492 vs. 9711 ± 1341, *p* = 0.02), respectively (Figure 5B). However, the binding of AAL to PBMCs was significantly lower in the case of CD11+ myeloid cells (14,357 ± 939 vs. 16,998 ± 3083, *p* = 0.02) (Figure 5A). The binding of SNA to the sialic acid moiety was significantly higher on the CD3+ T cells and CD11b+ myeloid cell surface of mice with CRC versus healthy controls (CD3+, 19,477 ± 10,632 vs. 11,958 ± 843, *p* = 0.05; CD11b+, 24,994 ± 8572 vs. 12,431 ± 1011, *p* = 4.7 × 10^−4^), respectively. PWM, N-Acetylglucosamine binding lectin showed significant differences in binding to cell surfaces. PWM binding was significantly higher in the case of the spleen and blood on CD11b+ myeloid cells. (6846 ± 294 vs. 5364 ± 533, *p* = 1.8 × 10^−6^, 6770 ± 516 vs. 5742 ±271, *p* = 7.4 × 10^−5^), respectively (Figure 5A,B). The binding of siglec-1 to sialic acid was significantly lower on the CD11b+ myeloid cell surface (12,599 ± 1593 vs. 18,051 ± 5151, *p* = 7.8 × 10^−3^), but siglec-1 binding was significantly higher (18,470 ± 4423 vs. 12,139 ± 692, *p* = 6.2 × 10^−4^) on the CD19+ B cells surface of spleen-derived cells vs. controls, respectively (Figure 5B). N-acetyllactosamine binding galectin-1 was significantly lower on the CD11b+ myeloid cells of the blood (113,547.2 ± 41,454.99 vs. 154,158.5 ± 28,484.31) (Figure 5A).

**Figure 6:**
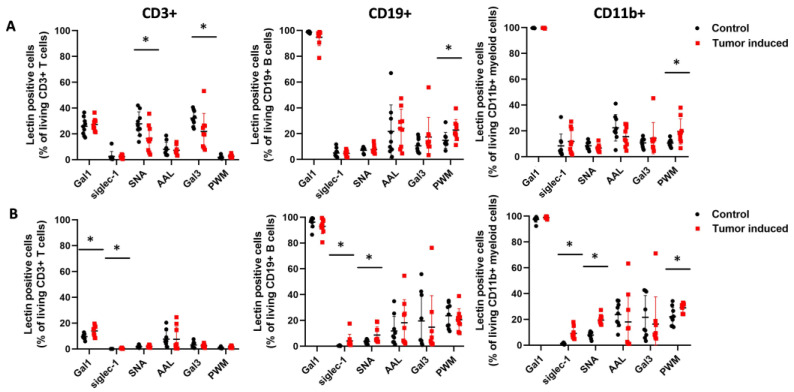
The percentage of lectin-reactive cells within CD3+ T cells, CD19+ B cells, and CD11b myeloid cells from peripheral blood (**A**) and the spleen (**B**) of CRC-bearing and heathy control mice analyzed by flow cytometry. Siglec-1 binding cell numbers were significantly higher in all three types of immune cells in the case of the spleen. SNA binding cell number was significantly higher in CD11b+ and CD19+ cells from the spleen but was significantly lower in CD3+ T cells of PBMC samples. PWM binding myeloid cells and lymphocytes showed a significantly increased number of cells compared to the healthy control. Arithmetic mean values and standard deviations (error bars) are shown, (n = 9 CT 26-bearing, n = 9 control). Flow cytometry statistical analysis was performed using a two-tailed, heteroscedastic Student’s *t*-test to evaluate the statistical significance (set at * *p* < 0.05) between two given experimental groups: pairwise comparison of each sample.

We also found significant differences in the percentage of reactive cells in three different types of immune cells of the CRC-bearing mice compared to healthy controls (Figure 6). The number of siglec-1 binding cells was significantly higher in all three types of immune cells in the case of spleen samples compared to the healthy controls (CD3+: 0.45 ± 0.34 vs. 0.037 ± 0.032, *p* = 2.5 × 10^−3^; CD11b: 9.11± 4.54 vs. 1.32± 0.66, *p* = 1.1 × 10^−4^; CD19+: 4.04± 5.23 vs. 0.4± 0.24, *p* = 0.05) (Figure 6B). Moreover, the SNA binding cell number was significantly higher at CD11b+ and CD19+ cells from the spleen of CRC-bearing mice vs. controls (19.49 ± 3.88 vs. 7.86 ± 2.51, *p* = 1 × 10^−6^ and 8.61 ± 6.46 vs. 3.58 ± 1.62, *p* = 0.03), but, in the case of PBMCs, the number of SNA reactive cells was significantly lower at CD3+ T cells of CRC-bearing mice compared to the healthy controls (16.5 ± 10.55 vs. 27.81 ± 9.23, *p* = 0.02) (Figure 6A,B). PWM binding CD11b+ myeloid cells and CD19+ B lymphocytes showed a significant increase in PBMCs of CRC mice compared to healthy controls (19.55 ± 9.80 vs. 10.53 ± 2.84, *p* = 0.01, 22.80 ± 8.17 vs. 14.89 ± 6.17, *p* = 0.03) (Figure 6A). Gal1 binding showed a significantly increased number of cells on CD3+ T cells in the case of spleen samples (13.92 ± 4.14 vs. 9.57 ± 2.13, *p* = 0.01); however, Gal3 binding was lower on CD3+ T cells of PBMC samples (21.91 ± 13.83 vs. 32.23 ± 5.89, *p* = 0.05) in mice with CRC vs. controls.

### 2.3. Proteomic Analysis of the Established Murine CT26 Tumor Tissues

**Figure 7:**
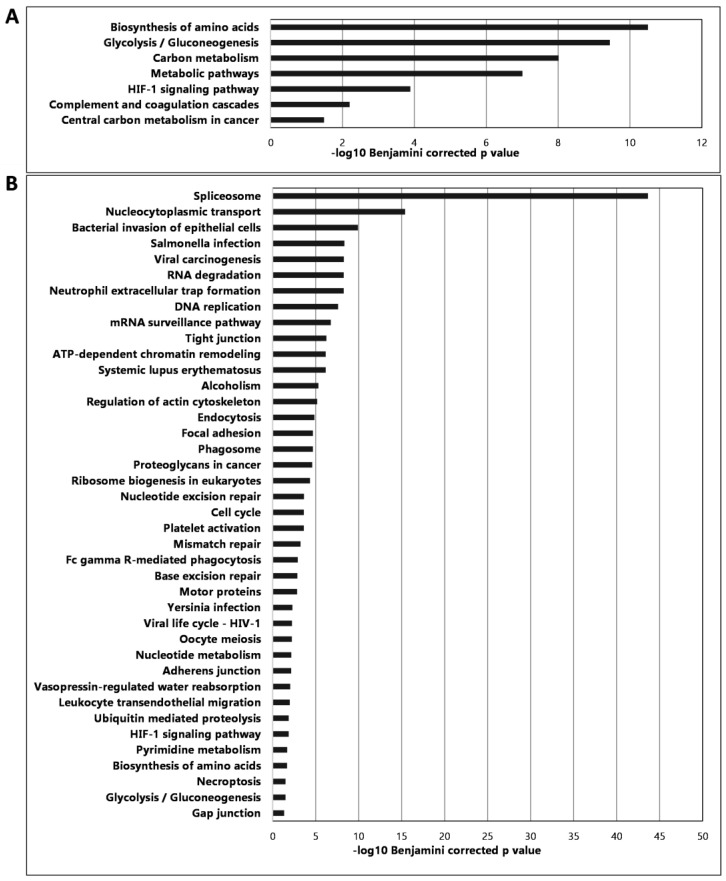
Proteomic analysis results showing the most significant pathways of the spleen (**A**) and the liver (**B**) showing the changes in CRC compared to healthy tissues.

To characterize the established murine CRC, the proteomic profiling of the established and excised primary CRC spleen and metastatic liver tissues was investigated by LC-MS in comparison with healthy spleen and liver spleen tissues, respectively. For whole proteome analysis, The Database for Annotation, Visualization and Integrated Discovery (DAVID) was used and KEGG (Kyoto Encyclopedia of Genes and Genomes) pathway analysis revealed that mostly metabolic traits were changed in CRC, such as biosynthesis of amino acids, glycolysis/gluconeogenesis, carbon metabolism, complement, and the coagulation cascade (Figure 7).

We paid special attention to the proteins involved in glycosylation (Supplementary Table S1) and we collected endogenous lectins that showed significant changes compared to healthy controls (Supplementary Table S2). LC-MS proteomic analysis showed 851 significant changes in the CRC-induced spleen versus the healthy control. From these, 574 proteins showed significantly decreased abundance and 277 increased significantly in the primary splenic tumors. In the metastatic liver, the total changes affected 2164 proteins, where 730 decreased and 1434 increased significantly in relation to the healthy control liver (Supplementary Tables S3 and S4). In the following tables, we have highlighted the top 10 differentially expressed proteins in the tumor-bearing spleen (Table 1) and metastatic liver (Table 2).

In order to validate the murine CT26 as a relevant CRC model, the LC-MS proteomic analysis of the primary and secondary tumor-bearing murine organs, the spleen and liver, was compared in silico with data of human CRC proteomics published by Chen et al. [46]. We could identify 99 proteins of the murine CT26-bearing spleen that showed coverage with the human CRC data set (Supplementary Table S5). We could also identify 154 proteins of the murine CT26-bearing liver that showed coverage with the human CRC data (Supplementary Table S6). However, there was a lack of perfect linear correlation in the overexpression or downmodulation of the identified proteins in the murine CT26 model compared with the human CRC data set; those 99 and 154 proteins were affected by colorectal cancer both in mice and human patients.

## 3. Discussion

The importance of animal models of CRC is essential because colon carcinoma is the third most common cancer worldwide resulting in a high percentage of mortality because of liver metastasis. The injection of CT26 into the spleens of BALB/c mice provides a reproducible syngeneic animal model within two weeks, which is comparable to human tumors, and the tumor growth mimics the characteristics of CRC, as well as the possible hepatic colonization of colorectal cancer spread to the liver [14,47]. Therefore, we had the opportunity to study the glycosylation patterns of leukocytes and the whole proteome profile of primary and secondary tumors in a narrow time frame.

Leukocytes are the central players in immunological processes. For example, the process of leukocyte adhesion has an important role in chronic inflammation and metastasis [48]. We found that the binding of AAL to fucose/arabinose structures was higher on the cell surface of T, myeloid CD11b+, and B cells of the mouse spleens with induced CRC versus healthy controls. Fucosylation is the most important process in leukocyte adhesion. It has been shown that defects in the fucosylation of glycoconjugates are due to leukocyte adhesion deficiency (LAD) [49]. Sialic acid moieties on leukocytes also are well-known modifiers of immune cell behavior. For example, surface α 2-6 sialic acids act as negative regulators of B cell activation [50]. Furthermore, it has been shown that hematopoietic stem cells have a high degree of α 2-6 sialic acid coverage on their cell surface, which decreases if they become more differentiated [51]. Therefore, following changes in SNA binding could be an essential diagnostic tool in any immunological process or cancer. The other sialic acid binding candidate lectin is siglec-1, which binds to α 2-6 sialic acid moieties. Hypersialylation participates in altered tumor cell adhesion and invasion via integrins, which cause the increased spread of tumor cells. In addition, due to the sialylation of cancer cells, they can also evade cell death pathways. Moreover, sialoglycans on the hypersialylated cancer cell surface bind to siglecs on immune cells to mediate immunosuppression to promote continued tumor growth [21]. Therefore, this could be an explanation for the significantly higher number of siglec-1 binding cells in the spleens of CRC-bearing mice. Moreover, Zhuo et al. have shown that the α 2-6-sialylation of β1 integrins in human colon carcinoma impairs adhesion to extracellular Gal3 and confers a selective advantage by protecting the tumor against Gal3-induced apoptosis [52]. On the other hand, we found that N-Acetylglucosamine (GlcNAc) binding PWM lectin shows a higher median intensity of binding, as well as a higher number of binding cells. Increased levels of GlcNAc in cancer have been shown in the literature [53]. For example, Annexin A1 shows increased levels of GlcNAcylation in colon cancer patients [54]. Annexin A1 is a calcium-dependent phospholipid-binding protein that interacts mainly with intracellular phospholipidic components and that has been associated with oncogenesis, vesicle, and membrane aggregation, exo- and endocytosis, mitosis, apoptosis, leukocyte migration, and activation. Last but not least, we chose N-Acetyllactosamine (LacNAc) binding galectins to investigate. It has been shown that the level of the beta-galactose beta1,3-N-acetylglucosaminyltransferase (B3GNT8) enzyme, which is an enzyme of poly-N-acetyllactosamine biosynthesis and synthesizes poly-N-acetyllactosamine, showed an increased level in colorectal carcinoma compared to normal tissues [55]. A previous study has shown that galectin-3 can bind to the LacdiNAc group on macrophages and regulate the immune response [56]. LacNAc also may serve as a scaffold for further modification with sialic acid, fucose moieties, or LacdiNAc termini, which are cancer-associated carbohydrate antigens [57]. Moreover, there are many carbohydrate antigens that are used in current cancer diagnosis. For example, carcinoembryonic antigen (CEA) and carbohydrate antigen 19-9 (CA19-9) are the most common tumor-associated antigens of patients with rectal cancer and colorectal carcinoma [58]. CA 15-3 and CA 27-29 are used in the clinical diagnosis of breast cancer, while CA 125 is a biomarker of ovarian cancer. N-glycolylneuraminic acid (Neu5Gc) is a sialic acid molecule found in mammals, However, humans cannot synthesize it. However, Neu5Gc can accumulate in cancer tissues, e.g., from diet sources, and therefore anti-Neu5Gc antibodies might have potential cancer biomarkers. Leviatan Ben-Arye et al. described a sialoglycan microarray for the detection of Neu5Gc [59]. The following glycans, Tn (Thomsen-nouvelle), sTN (sialyl Tn), and T (Thomsen-Friedenreich), are aberrant O-glycans, which are antigens associated with cancer progression and metastasis as well. Dimeric Lewis antigens such as sialyl-dimeric Lewis x (sLex) are expressed on human granulocytes, monocytes, small subsets of lymphocytes, and a variety of cancer tissues. Glycans participate in nearly all immunological processes, including recognition, activation, and differentiation; thus, their roles are essential in inflammatory and cancer diseases. Congenital disorder of glycosylation (CDG) is a serious illness that corroborates the necessity of a correct glycosylation system. CDG is associated with developmental delays and physiological abnormalities that are often fatal, and patients who survive adulthood have immune defects that result in recurrent infections [60]. All things considered, lectin-based multi-parallel carbohydrate chain detection could be a useful, modern diagnostic tool for cancer diagnosis and prognosis. However, one limitation of our study should be mentioned: the tissue resident and infiltration leukocytes cannot be distinguished in our syngeneic wild type Balb/c model. Taken together, the demonstrated differences between the tumor-bearing mice and healthy controls shed light on the altered glycosylation pattern in CRC.

Because of the complexity of the development of colorectal cancer in humans, the importance of proper animal models is unequivocal. Here, the authors characterized a CT26 transplantation model, which is a syngeneic CRC model in two aspects: one is the glyco-immunology approach, and the other is the proteomics of primary and secondary CRC tumor-affected tissues. The tumor shapes the immune system and influences the polarization of immune cells to escape clearance. By understanding the sugar code, the difficult glycosylated structures on the surface of immune cells may help us to understand their plasticity in response to tumor-derived factors. The authors used fluorescently labeled lectins to monitor the alterations in the cell surface glycosylation of T cells, B cells, and myeloid cells in CRC-bearing mice. Indeed, CT26 injection into the spleen or CT26 colonization to the liver changed the lectin-based immunophenotype compared to healthy control samples. Flow cytometric analysis of the splenocytes revealed the increased binding of SNA, and AAL to CD3+T cells and CD11b myeloid cells; and increased siglec-1 and AAL binding to the CD19 B cells of the tumor-bearing mice.

Endogen lectins also have a crucial role in cancer; for example, C-type lectins take part in the lymphatic and hematogenous metastasis of tumors by interacting with carbohydrate ligands on tumor cells. C-type lectins, including selectins, mannose receptor (MR), and lymph node sinusoidal endothelial cell C-type lectin (LSECtin), facilitate metastasis by involvement in the circulation of tumor cells in the blood. A member of the MR family, the Endo180, is an endocytic receptor that interacts with glycosylated collagens and binds to N-acetylglucosamine. Endo180 is expressed on stromal cells and thus takes part in intracellular collagen degradation. This process is responsible for tissue remodeling, tumor invasion, and metastasis. It has been shown that patients with breast cancer with elevated levels of serum Endo180 exhibit increased metastatic potential [61]. For that reason, we thought that performing complete proteomics could provide an even more comprehensive picture of the role of lectins in cancer. After whole proteome analysis, we found significant differences in a lot of glycosylation enzymes and endogenous lectins in CRC-bearing animal tissues compared to healthy controls. Glycosyltransferases, galactosyltransferases, mannosyltransferases, and fucosyltransferases showed a significantly higher fold change in the spleen and in the liver in tumor-bearing mice (Supplementary Table S1). In addition, we found several significantly increased amounts of endogenous lectins, such as collectin 12, sialoadhesin, galectin-1, galectin-3, galectin-7, and C-type mannose receptor 2 in the CRC spleen and liver compared to the healthy control. However, galectin-9 showed a decreased amount of the CRC-bearing mice’s spleen and liver too (Supplementary Table S2). Most of the CT26-bearing spleen-derived proteins were involved in amino acid biosynthesis, glycolysis-carbon metabolism, and hypoxia pathways. Most of the CT26-bearing liver-derived proteins were involved in transcription/translation, stress responses like viral infections, and cell division.

The in silico comparison of our murine CT26-bearing spleen and liver proteomics data with human CRC proteomics revealed 99 and 154 proteins that were affected both in primary and secondary tumor development as common variables in murine or human CRC. However, the identified proteins were affected by CRC both in our mouse model and in human samples, but there were no perfect correlations of murine and human proteomics data in terms of overproduction or downmodulation. The induction of some proteins that were overexpressed in our CT26-bearing spleen model has been previously reported and it led to a poor prognosis in colorectal patients, such as Carbonic anhydrase IX (fold change tumor/control: FC: 26.155) [62,63], protein S100A4 (FC: 23.44) [64], and galectin-1 (FC: 10.25) [65,66]. The protein S100A4 (FC: 60.8) and the galectin-1 (FC: 54.7) were also upregulated in CT26-bearing liver tissue. Additionally, the following proteins were previously reported to contribute to colorectal cancer progression and poor prognosis and were induced in the secondary CT26 tumor-bearing livers: galectin-3 (FC: 73.4) [67], proliferation marker protein Ki-67 (FC: 37.3) [68], and the High Mobility Group Protein 1 (FC: 25.7) [69]. Taken together, the in silico comparison of our CT26 murine model with human CRC verified the usefulness and applicability of our model for CRC research.

The strengths of our study are the following: (1) An in vivo model was established instead of performing only in silico data analysis. (2) Single-cell immunophenotyping was performed comparing the T, B cells, and myeloid cells of CT26 CRC-bearing mice with healthy controls. (3) The single cell immunophenotyping was performed not only by antibodies but fluorescently conjugated lectins were also used as a tool kit to monitor cell surface glycosylation of the immune cells. (4) The proteomics study of the primary (spleen) and secondary (liver) CT26 tumors identified proteins that were affected also in a human CRC data set (Chen et al.). Additionally, S100A4 and galectin-1 were identified as overexpressed both in the murine CT26-bearing spleen and liver, and these were previously reported to contribute to disease progression and poor prognosis in human CRC patients.

Limitations of our study also should be mentioned. Although the authors revealed alterations in the cell surface glycosylation of immune cells, as the tumor-driven change in the sugar code (glyco-immunophenotype), and the authors identified 851 and 2164 differentially expressed proteins in the primary or secondary CT26-bearing organs, the authors could not address or identify the CT26-derived soluble factors that shaped the immunophenotype and altered the cell surface glycosylation of the immune cells. Additionally, the authors could not address the functional consequence of the altered glyco-immunophenotype, whether it caused immunosuppression, T cell anergy, or in the opposite, whether it was associated with the activation of these immune cells to combat cancer. To answer these questions, further mechanistic studies should be carried out.

## 4. Materials and Methods

### 4.1. Animals

Mice were kept in a sterile environment in Makrolon^®^ cages at 22–24 °C (40–50% humidity), with light regulation of a 12/12 h light/dark cycle. The animals had free access to tap water and were fed a sterilized standard diet (VRF1, autoclavable, Akronom Kft., Budapest, Hungary) ad libitum. Animals used in our study were taken care of according to the “Guiding Principles for the Care and Use of Animals” based on the Helsinki Declaration, and they were approved by the ethical committee of the National Institute of Oncology. Animal housing density was assessed according to the regulations and recommendations from directive 2010/63/EU of the European Parliament and the Council of the European Union on the protection of animals used for scientific purposes. The permission licenses for breeding and performing experiments with laboratory animals were PEI/001/1738-3/2015 and PE/EA/1461-7/2020.

### 4.2. Cell Lines and Tissue Culture Conditions

CT-26 cells were obtained from ATCC and cultured in RPMI-1640 Medium (BioSera, Boussens, France), supplemented with 10% fetal bovine serum (BioSera, Boussens, France) and 1% Penicillin/Streptomycin (BioSera, Boussens, France) in sterile T75 tissue culture flasks with ventilation caps (Sarstedt, Nümbrecht, Germany) in a humidified 5% CO_2_ atmosphere at 37 °C. Cells were cultured for a maximum of 25 passages or 60 days after thawing and were screened for mycoplasma infection.

### 4.3. Spleen Injection

Single-cell suspensions were prepared from CT-26 monolayer cultures, washed with PBS (BioSera, Boussens, France), and diluted in Medium 199 (Lonza, Basel, Switzerland). Overall, 2 × 10^3^ tumor cells were injected in a volume of 50 μL into the spleens of adult male inbred Balb/c mice from where metastatic colonies formed in the liver (n = 9 CT26 injected, n = 9 vehicle injected control). Under anaesthetization, the skin was shaved and rubbed with ethanol pads. Next, a 6–8 mm incision was made adjacent to the spleen (a left flank incision approximately 2 cm left of the abdominal midline). The spleen was gently pulled out of the abdominal cavity and 50 µL of cell suspension was injected into the lower third of the spleen. After that, the spleen was carefully reinserted, and the abdominal wall and skin were surgically closed. Control animals were injected with 50 μL medium. The treated animals were sacrificed on day 22 after tumor inoculations. Tumors were evaluated macroscopically for immunohistochemistry.

### 4.4. Immunohistochemistry

The IHC was performed as described previously by our group with some modifications [70,71]. Briefly, mice were sacrificed at 22 days. Livers and spleens were removed and fixed overnight in 4% formalin, then embedded in paraffin, and cut into 3 μm sections with a Rotary microtome (RM2235, Leica, Wetzlar, Germany). Immunohistochemistry was performed on the platform of the Leica company, BOND-MAX Immunohistochemical staining machine. The primary antibodies used for immunohistochemistry are anti-arginase-1 (GenomeMe, IHC400-100, 1:800, Richmond, BC, Canada), anti-CD45 antibody (Biocare, CM016, 1:200, Warrington, UK), anti-CDX2 antibody (Cellmarque, 235R-15, 1:200, Rocklin, CA, USA), anti-Pan-Cytokeratin antibody (Cellmarque, 313M-14, 1:600, Rocklin, CA, USA), and anti-Vimentin antibody (Novocastra, NCL-L-VIM-V9, 1:500, Deer Park, IL, USA) with overnight incubation. A labeling system (Bond Polymer Refine Detection, DS9800, Leica) containing secondary antibodies labeled with horseradish peroxidase (HRP) and DAB-3 (3′-diaminobenzidine) was used as the chromogen for antigen signal detection. Hematoxylin (ready to use, Leica) was used for contrast staining. The Zeiss Axio Imager Z1 microscope (ocular 10×, objectives 10×, 40×) was used for visualization with the Zeiss AxioCam MRm camera and AxioVision SE64 4.9.1 software (Carl Zeiss AG, Oberkochen, Germany).

### 4.5. Peripheral White Blood Cell Isolation

Leukocytes were isolated as described previously (n = 9 CT26 injected, n = 9 vehicle injected control) [45]. Briefly, they were taken freshly from the blood of mice via cardiac puncture. EDTA-treated whole blood was centrifuged at 400× *g* for 10 min, and the supernatant was removed. Red blood cell lysis was carried out by the incubation of the cells with 5 mL ACK buffer (0.155 M NH_4_Cl, 10 mM KHCO_3_, 0.1 mM Na_2_EDTA, pH 7.3, GIBCO, Cat. A1049201, Thermo Fischer Scientific, New York, NY, USA) solution at RT for 5 min. Samples were loaded on a cell strainer (70 μm in pore size) and washed twice with 10 mL PBS. Cells were resuspended and pipetted into 12 × 75 mm FACS tubes (VWR International, Radnoe, PA, USA) and diluted with 100 µL staining buffer (PBS with 1% FBS, GIBCO, Life Technologies, Paisley, UK, and 0.1% sodium azide, Sigma-Aldrich, Saint Louis, MO, USA).

### 4.6. Spleen Cell Isolation

The spleen was processed freshly as described previously with minor modifications (n = 9 CT26 injected, n = 9 vehicle injected control) [72]. Briefly, after the spleen was smashed on a 100 μm cell strainer, (VWR, Radnoe, PA, USA), washed with PBS, and centrifuged at 1400 rpm for 5 min, the pellet was resuspended in sterile PBS. Red blood cell lysis was carried out by the incubation of cells with 5 mL ACK solution for 5 min. Samples were loaded on a cell strainer (70 μm in pore size) and washed twice with 20 mL PBS. After that, cells were resuspended and pipetted into 12 × 75 mm FACS tubes (VWR International, PA, USA) and diluted with 100 µL staining buffer (PBS with 1% FBS, GIBCO, Life Technologies, Paisley, UK, and 0.1% sodium azide, Sigma-Aldrich, Saint Louis, MO, USA).

### 4.7. Lectin Labeling

For the fluorescent labeling of lectins, we used conjugation kits (Lightning-Link, Abcam, Cambridge, MA, USA) according to the manufacturer’s instructions. Aleuria Aurantia lectin (AAL, Cat. L-1390-2, Vector Laboratories, Newark, NJ, USA) was labeled with APC (ab201807, Lot. GR3388854-1), Sambucus nigra lectin (SNA, Cat. 21510104 Glycomatrix, Dublin, OH, USA) was labeled with PE/Cy7 (ab102903, Lot. GR3390407-1), human sialoadhesin, siglec-1/CD169 recombinant protein (siglec-1, Cat. 5197SL050, Fischer Scientific, Waltham, MA, USA) were labeled with PE/Texas Red (ab269899, Lot. GR3395603-2), galectin-1 (Gal-1, Cat. 450-39, PreproTech, London, UK) was labeled with Alexa Fluor 488 (ab236553, Lot. GR3394511-1), galectin-3 (Gal3, 450-38, PreproTech, London, UK) was labeled with APC/Cy7 (ab102859, Lot. GR3396716-1), and Phytolacca americana lectin (PWM, Cat. L8777, Sigma-Aldrich, Saint Louis, MO, USA) was labeled with Alexa Fluor 700 (ab269824, Lot. GR3393759-2) dye by the lectin amine groups. The list of the selected lectins for cytometry is listed in Table 3. Before experiments, we optimized the concentration of antibodies and labeled lectins by titration on a flow cytometer (C40313, CytoFLEX LX N3-V5-B3-Y5-R3-I0, Beckman Coulter, IN, USA).

### 4.8. Lectin Agglutination Test

To obtain the best results, we had to know whether each lectin was capable of cell agglutination, and we had to exclude them from the experiments (Supplementary Figure S1). After euthanizing the animals, the spleens were removed and homogenized freshly on a cell strainer (70 μm pore size, Merck Millipore, Burlington, MA, USA) using the piston of a syringe and sterile PBS. Cells were pelleted by centrifugation at 1500 rpm for 5 min. The pellet was resuspended in 5 mL ACK lysis buffer for 5 min. Samples were loaded on a cell strainer (70 μm in pore size) and washed with 20 mL PBS. Cells were counted using a Bürker chamber and trypan blue viability dye (Thermo Fischer Scientific, Waltham, MA, USA), and 100.000 cells were plated on 96-well plates (Corning, New York, NY, USA) for a lectin agglutination assay and microscopic evaluation (HoloMonitor™ M3). The agglutination assay consisted of four different concentrations (0,1 µg/mL; 1 µg/mL; 10 µg/mL and 50 µg/mL) in RPMI (GIBCO, Life Technologies, Paisley, UK). Lectins were diluted in sterile PBS with 0,1% sodium azide (Sigma-Aldrich, Saint Louis, MO, USA). The lectins involved in the study were used in a lower concentration than the concentration with the agglutination activity of leukocytes.

### 4.9. Labeling and Flow Cytometry

Cell surface staining was performed as described previously by our group with some modifications [73,74]. Briefly, the antibody and lectin cocktail for extracellular staining was added to the freshly isolated white blood cells in 100 µL staining buffer (PBS, 1% fetal bovine serum, Gibco, Life Technologies, Paisley, UK, 0.1% sodium azide, Sigma-Aldrich, Saint Louis, MO, USA). The fluorescently labeled lectins were titrated on healthy leukocytes in 2.5 µg/mL, 1 µg/mL, 0.5 µg/mL, and 0.1 µg/mL, as shown in Supplementary Figure S2. The antibody cocktail consisted of 80× dilutions of eFluor 450 anti-mouse CD3 (Cat. 48-0032-82, Clone 17A2, Thermo Fischer Scientific, Waltham, MA, USA), BV605 anti-mouse CD11b (Cat. 101237, clone M1/70, BioLegend, San Diego, CA, USA), BV650 anti-mouse CD19 (Cat. 115541, clone 6D5, BioLegend, San Diego, CA, USA), PerCP/Cyanine5.5 anti-mouse CD45 (Cat. 103132, Clone 30-F11), 100× dilution of AAL, Gal1, siglec-1, 10× dilution of SNA, 5 × dilution of PWM, Gal3, HPA, and a 1000× dilution of Viability fixable dye eFluor 506 (Cat. 65-0866-14, Thermo Fischer Scientific, Waltham, MA, USA). Cells were incubated at 4 °C for 30 min. All antibodies were used according to the manufacturer’s instructions. Cells were washed with 500 µL PBS and centrifuged at 500× *g* for 5 min. Cells were resuspended in 400 µL PEB and were measured on Cytoflex LX fluorescence-activated cell sorter (C40313, CytoFLEX LX N3-V5-B3-Y5-R3-I0, Beckman Coulter, IN, USA). Manual gating was used to determine CD3+, CD19+, and CD11b+ cells within singlet living cells in CytExpert (Beckman Coulter). Unstained cells were used as an absolute negative control. The gating strategy for flow cytometry is shown in Supplementary Figure S3. Lectin-binding positive cells were gated manually within CD3+, CD19+CD3-, and CD11b+CD3-CD19-. Negative control samples were incubated with antibodies and viability dye but lacked fluorescently labeled lectins. At least 10^6^ gated events were acquired using CytoFLex LX and analyzed manually using the CytExpert v.2.4.0.28 software (Beckman Coulter, IN, USA).

### 4.10. Proteomic Characterization

Spleen (n = 8 tumor-bearing and n = 8 healthy spleen) tissue samples, and liver (n = 8 metastatic and n = 8 healthy liver) samples were homogenized in 10% SDS/50 mM TEAB using a TissueLyser (Qiagen, Hilden, Germany). The resulting lysates were centrifuged (14,000 rcf for 10 min), the protein content of the supernatant was determined by the BCA colorimetric assay (Thermo Scientific), and 100 µg protein was digested according to the S-Trap mini protocol (https://protifi.com/pages/protocols, accessed on 22 February 2024). Briefly, protein disulfide bridges were reduced using TCEP (Tris(2-carboxyethyl)phosphine) and free thiol groups blocked by MMTS (S-methyl methanethiosulfonate) followed by 2 h digestion with sequencing-grade trypsin (2.5 µg/sample) at 47 °C. The resulting peptide mixtures were dried down and then labeled with TMTpro 16plex isobaric tags (Thermo Scientific) following the producer’s protocol. Samples were combined in a 1:1 ratio and fractionated off-line using a high-pH reversed-phase peptide fractionation kit (Thermo Scientific) according to the producer’s protocol. Fractions were combined to yield four final fractions (fractions 1 + 5, 2 + 6, 3 + 7, 4 + 8), vacuum-dried, and approximately 1 µg of total peptide amounts was loaded onto C18 EvoTips (Evosep) for LC-MS analysis. Reversed-phase separation of the peptides was performed using an Evosep One HPLC (Evosep) applying the “15 SPD” 88-min gradient followed by SPS-MS3 data acquisition using an Orbitrap Fusion Lumos Tribrid (Thermo Scientific) instrument equipped with an FAIMS Pro ion mobility device. Data were collected using two compensation voltages (−70 and −50 V) in alternating 1.5 s cycles. High-resolution MS3 data were generated from the top 6 most abundant fragment ions isolated simultaneously from each CID MS2 acquired in the linear ion trap.

Peptide identification and quantitation were performed using the Proteome Discoverer software (v3.0). Peptides and proteins were identified with the Sequest HT search engine using the mouse subset of the UniProt protein database (v2023-08-07, 17734 sequences). Relative quantitation was performed using the TMT reporter ion signal-to-noise (S/N) values extracted from MS3 HCD spectra. Unique and razor peptides were considered for the protein abundance-based comparison of the tumor and control sample groups (acceptance parameters: Sequest HT Xcorr > 1, precursor ion co-isolation threshold in MS1 <50%, average TMT reporter S/N in MS3 HCD spectra > 10, SPS mass matches in MS2 >50%). Proteins identified with high confidence showing |log2FoldChange| > 1 (adjusted *p*-Value ≤ 0.05) were accepted as differently expressed. The maximum and minimum fold changes assigned by the software were 100 and 0.01, respectively. Functional analysis: Genes corresponding to differentially expressed proteins (proteins identified with high confidence showing |log2FoldChange| > 1 and adjusted *p*-Value ≤ 0.05) were subjected to KEGG pathway enrichment analysis using the DAVID (The Database for Annotation, Visualization and Integrated Discovery) platform [75]. The KEGG pathway terms with Benjamini–Hochberg corrected *p*-Value < 0.05 and fold enrichment > 2 were accounted for significance.

### 4.11. Data Visualization and Statistics

GraphPad Prism software (version 8.0.1 for Windows, GraphPad Software) was used for FACS data visualization. For flow cytometry, statistical analysis was performed using a two-tailed, heteroscedastic Student’s *t*-test to evaluate the statistical significance (set at * *p* < 0.05, ** *p* < 0.01, *** *p* < 0.001) between two given experimental groups: pairwise comparison of each sample.

## Figures and Tables

**Table 1:** Proteomic analysis results of the CT26 tumor-bearing spleen highlighting the 10 most significantly decreased or 10 most significantly increased proteins in the tumor. The complete list and statistics are found in Supplementary Table S3.

Accession Number (Uniprot)	Description	Fold Change (Tumor/Control)
P19437	B-lymphocyte antigen CD20	−38.46
Q91WN4	Kynurenine 3-monooxygenase	−31.25
Q62507	Cochlin	−26.32
P42209	Septin-1	−25.64
P11911	B-cell antigen receptor complex-associated protein alpha chain	−24.39
P04224	H-2 class II histocompatibility antigen, E-K alpha chain	−22.22
Q8BV49	Pyrin and HIN domain-containing protein 1	−21.74
Q99JY3	GTPase IMAP family member 4	−20.41
Q9ESM6	Glycerophosphoinositol inositolphosphodiesterase GDPD2	−20
P11032	Granzyme A	−19.61
Q8VHB5	Carbonic anhydrase 9	26.16
P06837	Neuromodulin	27.13
P30204	Macrophage scavenger receptor types I and II	27.82
P22935	Cellular retinoic acid-binding protein 2	35.85
P0DOG8	Glyco-Gag protein	43.60
P52927	High mobility group protein HMGI-C	46.88
P70202	Latexin	56.80
P52624	Uridine phosphorylase 1	65.19
E9Q7T7	Chondroadherin-like protein	68.25
Q64437	All-trans-retinol dehydrogenase [NAD(+)] ADH7	100

**Table 2:** Proteomic analysis results of the CT26 metastatic livers highlighting the 10 most significantly decreased or 10 most significantly increased proteins in the tumor. The complete list and statistics are found in Supplementary Table S4.

Accession Number (Uniprot)	Description	Fold Change (Tumor/Control)
P11589	Major urinary protein 2	−55.56
P20852	Cytochrome P450 2A5	−27.03
P04939	Major urinary protein 3	−25.64
P00186	Cytochrome P450 1A2	−21.74
O09158	Cytochrome P450 3A25	−21.74
Q64176	Carboxylesterase 1E	−21.74
Q64459	Cytochrome P450 3A11	−17.24
Q8R1S9	Sodium-coupled neutral amino acid transporter 4	−16.95
Q64458	Cytochrome P450 2C29	−15.87
Q571F8	Glutaminase liver isoform, mitochondrial	−15.15
Q9R1Q7	Proteolipid protein 2	68.83
P16110	Galectin-3	73.50
P97310	DNA replication licensing factor MCM2	81.89
Q8K009	Mitochondrial 10-formyltetrahydrofolate dehydrogenase	82.70
Q61753	D-3-phosphoglycerate dehydrogenase	100
P25206	DNA replication licensing factor MCM3	100
P52927	High mobility group protein HMGI-C	100
P14069	Protein S100-A6	100
P70202	Latexin	100
Q64437	All-trans-retinol dehydrogenase [NAD(+)] ADH7	100

**Table 3:** List of the selected lectins for cytometry.

Lectin	Abbreviations	Preferred Sugar Specificity	Fluorescent Dye
*Phytolacca americana* lectin	PWM/PWA	N-Acetylglucosamine	Alexa Fluor 700
*Aleuria aurantia* lectin	AAL	Fucose, Arabinose	APC
*Sambucus nigra* lectin	SNA/EBL I	α 2-6 Sialic Acid	PE/Cy7
Galectin-1	Gal1	N-acetyllactosamine	Alexa Fluor 488
Galectin-3	Gal3	N-acetyllactosamine	APC/Cy7
Sialoadhesin	siglec-1/CD	α 2-3 Sialic Acid	PE/Texas Red

## Data Availability

Raw data files are available from the corresponding authors upon request.

## Supplementary Materials

The following supporting information can be downloaded at https://www.mdpi.com/article/10.3390/ijms25074022/s1.
