# Supplementary material for: Lectin-Based Immunophenotyping and Whole Proteomic Profiling of CT-26 Colon Carcinoma Murine Model
Source: Int J Mol Sci. 2024 Apr 4;25(7):4022. doi: 10.3390/ijms25074022 (PMC11012250; doi:10.3390/ijms25074022)
Supplement: Supplementary file 1 [file ijms-25-04022-s001.zip › Supplementary_Figure_Legend.pdf]

Supplementary Figures 1: Representative images about lectin agglutination test. High lectin concentration (50 µg/ml) can cause cell agglutination, especially in the case of MAA (Maackia amurensis Lectin), PSA (Pisum sativum lectin), LCA (Lens culinaris lectin). Therefore, we excluded these from the further investigations. (Scale bar 100 µm, magnification scale: 40X).

Supplemetary Figures 2: Titration of lectins for flow cytometry. After lectin labeling we performed a four step dilution titration of labeled lectins for each dye to choose the useable concentration.

Supplementary Figures 3: The gating strategy of flow cytometry. One million events were gated in the first „cells" population, later „singlets", and „living singlets" ere determined. CD45- negative (non-adherent) cells as immature hematopoietic cells, and CD45+ cells were gated as matured hematopoietic cells, furthermore CD3+ T-cells, CD19+ B-cells, and CD11b+ myeloid cells were gated within the CD45+ living singlets. Each main population (T-cells: green, B-cells: red, and myeloid cells: blue colors) was gated manually as lectin-positive(A) comparing to control lectin-negative(B) populations for all 6 lectins. Lectin-negative samples were stained with only anti-CD markers and viability dye but lacking fluorescently labeled lectins.

Supplementary Table 1: Representative table of significant changes of enzymes taking part in glycosylation processes in spleen and liver. Basic glycosylation enzymes (red frame) showed increased fold change in CRC spleen and liver compared to healthy control tissues.

Supplementary Table 2: Representative table of significantly changed endogenous lectins in CRC tissues compared to the corresponding healthy tissue. Galectin-1, galetin-3, and C-type mannose receptor 2 showed significantly increased amount in both the liver and spleen, however, galectin-9 decreased significantly in both cancerous organs compared to healthy controls.

Supplementary Table 3: Proteomic analysis results of the CT26 tumor bearing spleen in relation to control healthy spleen. LC-MS was performed as described the the Materials and Methods section. (n=8 for tumor bearing spleen and n=8 for healthy spleen).

Supplementary Table 4: Proteomic analysis results of the CT26 metastatic livers in relation to control healthy liver. LC-MS was performed as described the the Materials and Methods section. (n=8 for metastatic liver and n=8 for healthy liver).

Supplementary Table 5: In silico comparison of the proteomics of murine CT26 tumor bearing spleen with human CRC (Chen et al, PMID: 34590603). The column E lists 99 proteins ((gray background) of murine CT26 bearing spleen-related LC-MS results that showed coverage with human CRC proteomics data set. Right panel corresponds to the human CRC data. Abbreviations: RMC = right-sided mucinous colon cancer, NC = Normal colonic tissue, RNMC=right-sided non-mucinous colon cancer, LMC=left-sided mucinous colon cancer, LNMC=left-sided non-mucinous colon cancer

Supplementary Table 6: In silico comparison of the proteomics of murine CT26 tumor bearing liver with human CRC (Chen et al, PMID: 34590603). The column E lists 154 proteins (gray background) of murine CT26 bearing liver-related LC-MS results that showed coverage with human CRC proteomics data set. Right panel corresponds to the human CRC data. Abbreviations: RMC = right-sided mucinous colon cancer, NC = Normal colonic tissue, RNMC=right-sided non-mucinous colon cancer, LMC=left-sided mucinous colon cancer, LNMC=left-sided non-mucinous colon cancer
