# Supplementary figures and images for: Lectin-Based Immunophenotyping and Whole Proteomic Profiling of CT-26 Colon Carcinoma Murine Model
Source: Int J Mol Sci. 2024 Apr 4;25(7):4022. doi: 10.3390/ijms25074022 (PMC11012250; doi:10.3390/ijms25074022)

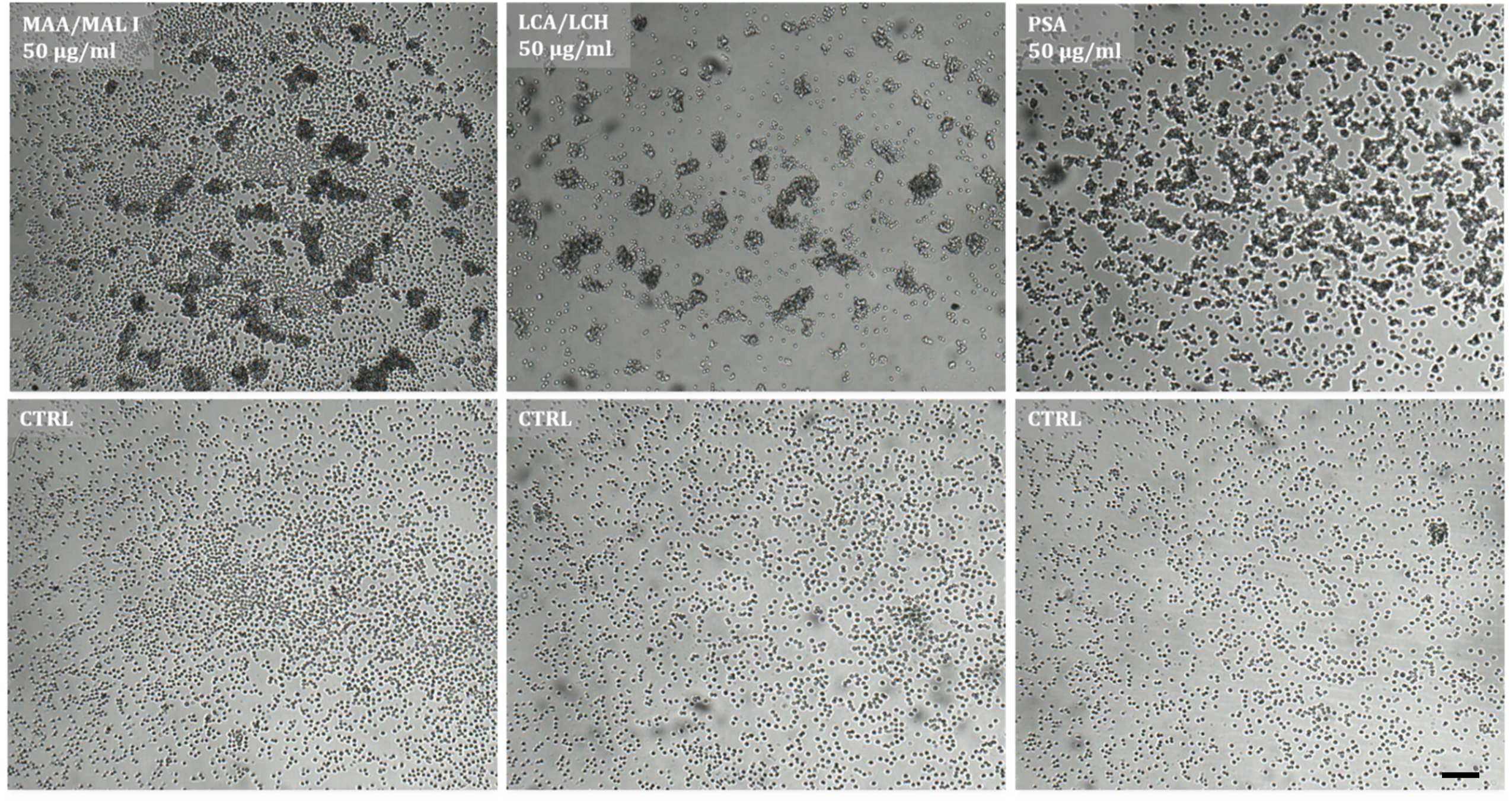

Supplement: Supplementary file 1 [file ijms-25-04022-s001.zip › SupplementaryFigure1.tif]

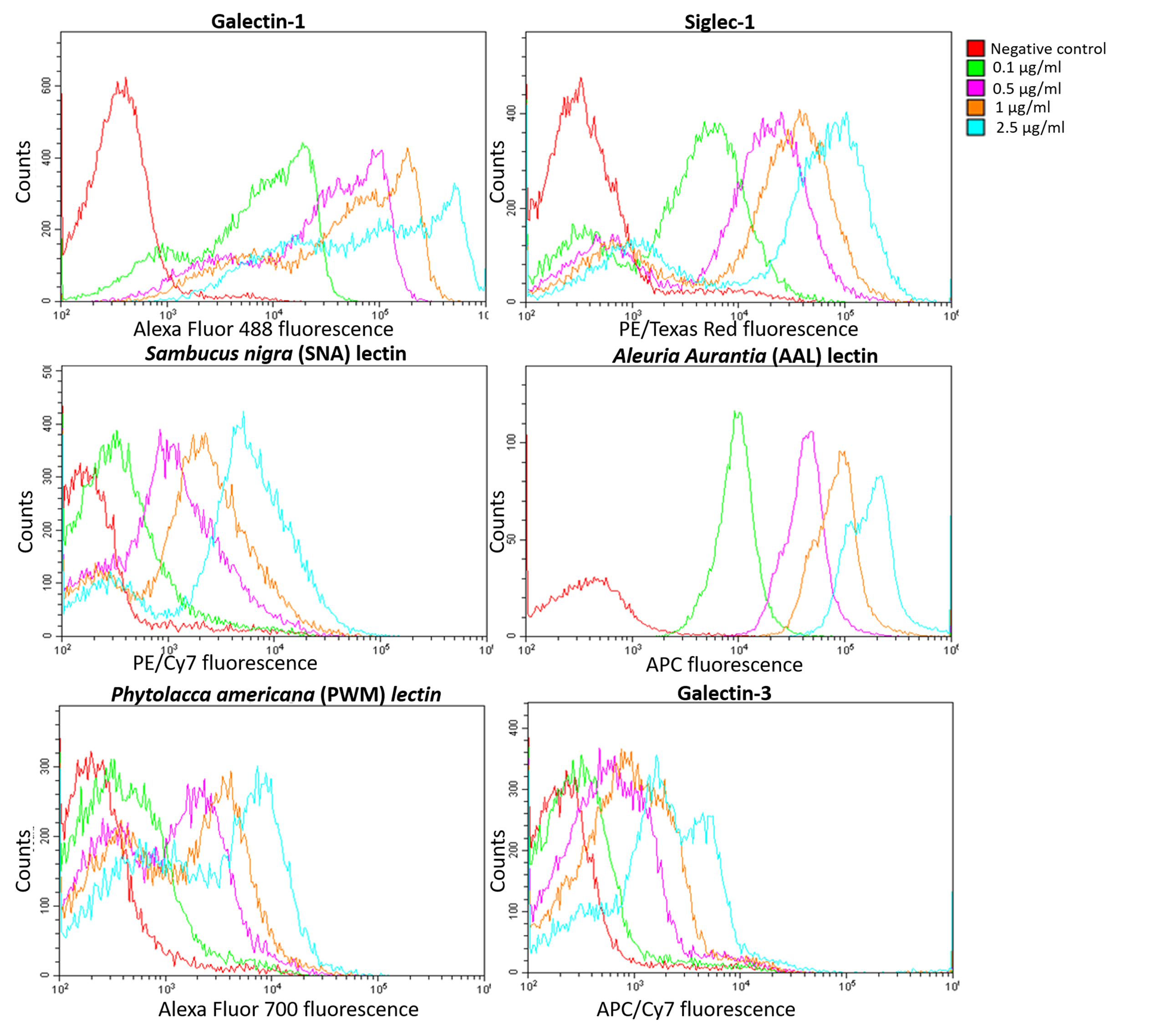

Supplement: Supplementary file 1 [file ijms-25-04022-s001.zip › SupplementaryFigure2.tif]

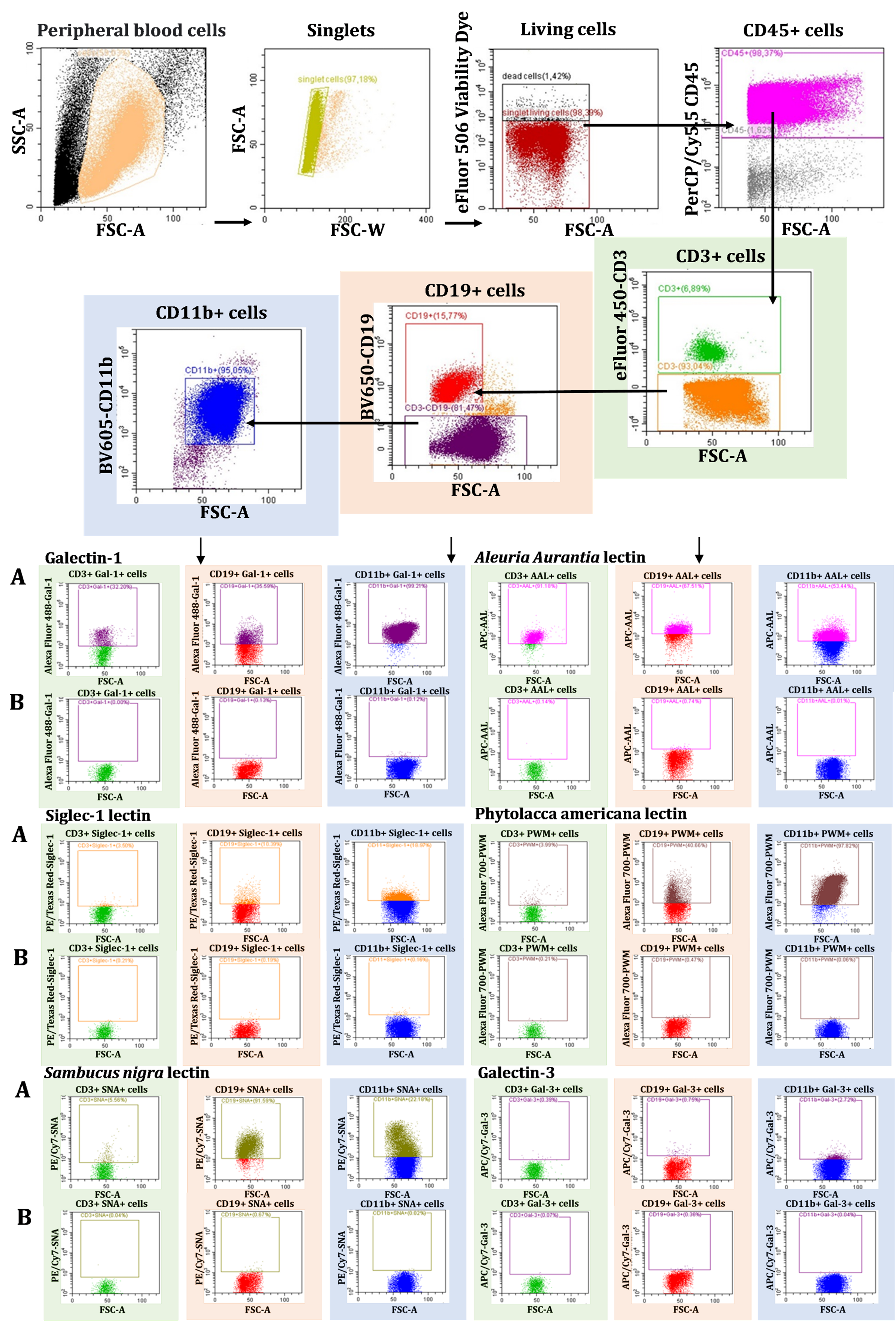

Supplement: Supplementary file 1 [file ijms-25-04022-s001.zip › SupplementaryFigure3.tif]

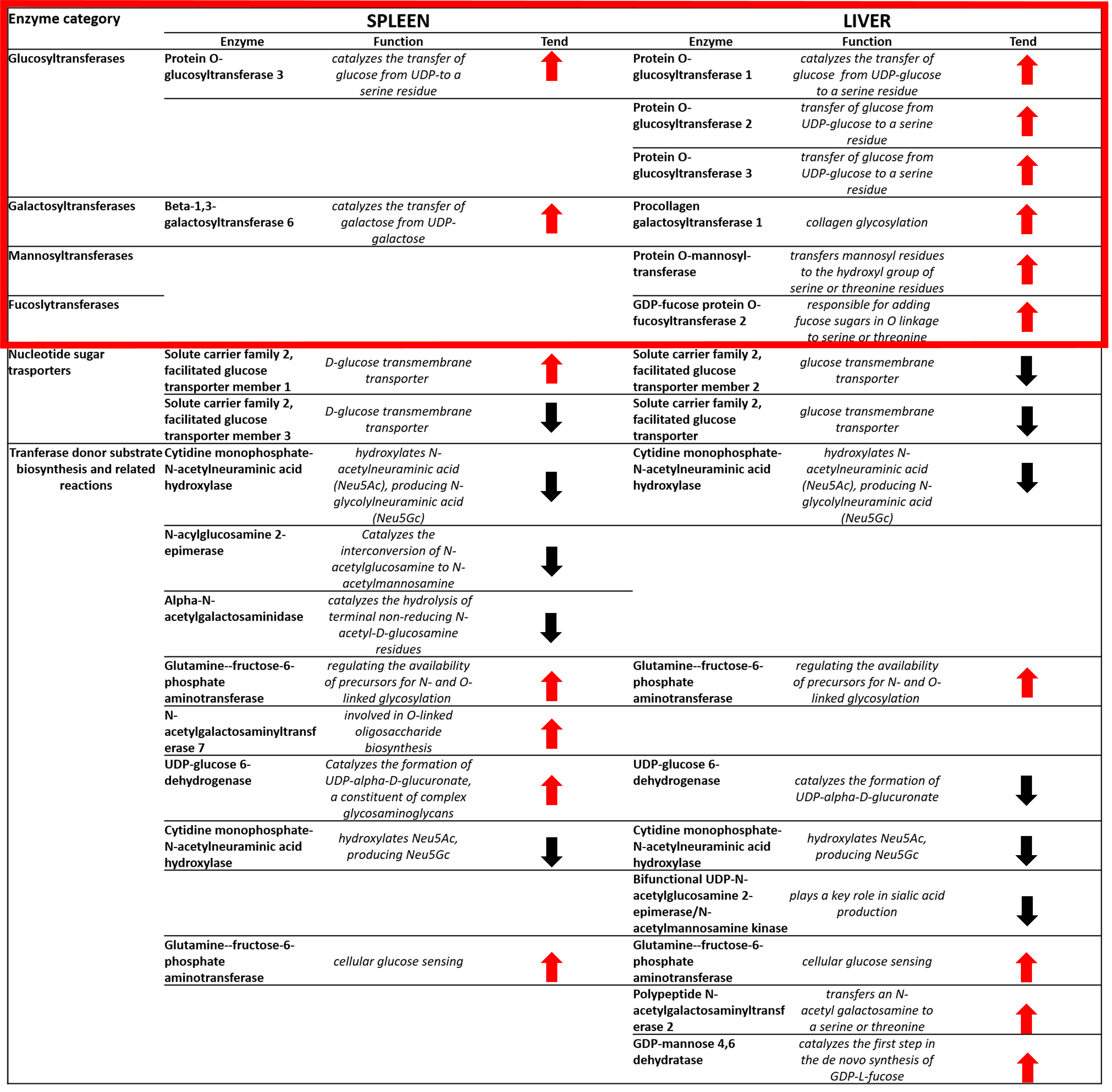

Supplement: Supplementary file 1 [file ijms-25-04022-s001.zip › SupplementaryTable1.tif]

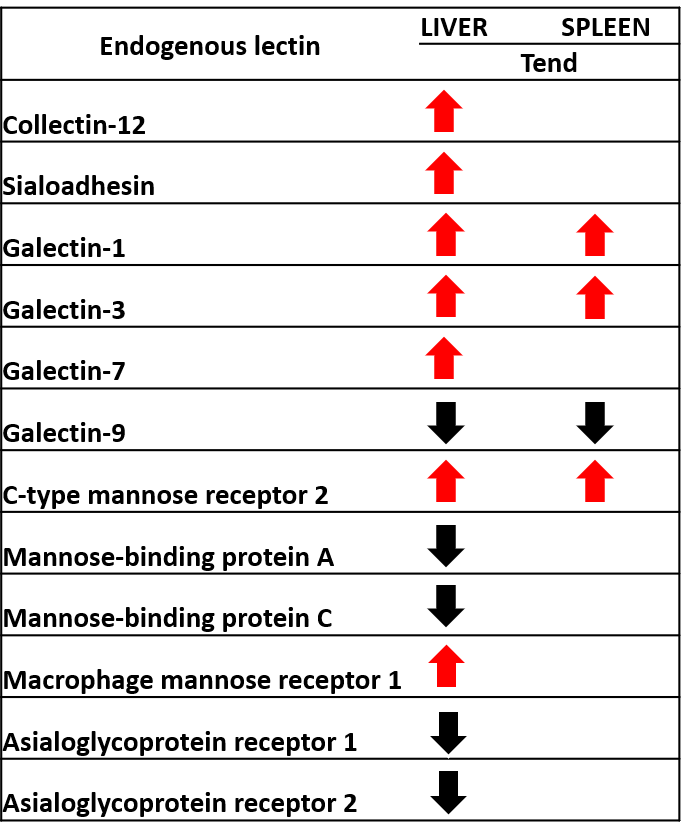

Supplement: Supplementary file 1 [file ijms-25-04022-s001.zip › SupplementaryTable2.tif]
